# Supplementary material for: Identification and functional analysis of non-coding regulatory small RNA FenSr3 in Bacillus amyloliquefaciens LPB-18
Source: PeerJ. 2023 May 15;11:e15236. doi: 10.7717/peerj.15236 (PMC10194069; doi:10.7717/peerj.15236)
Supplement: Supplemental Information 4 [file peerj-11-15236-s004.zip › KO/CK-vs-T1_map/map02060.html]

KEGG PATHWAY: Phosphotransferase system (PTS) - Reference pathway


|  |  |
| --- | --- |
| **Phosphotransferase system (PTS) - Reference pathway** |  |

[
Pathway menu
| Organism menu
| Pathway entry
| Show description
| User data mapping
]

The phosphoenolpyruvate (PEP)-dependent phosphotransferase system (PTS) is a major mechanism used by bacteria for uptake of carbohydrates, particularly hexoses, hexitols, and disaccharides, where the source of energy is from PEP. The PTS consists of two general components, enzyme I (EI) and histidine phosphocarrier protein (HPr), and of membrane-bound sugar specific permeases (enzymes II). Each enzyme II (EII) complex consists of one or two hydrophobic integral membrane domains (domains C and D) and two hydrophilic domains (domains A and B). EII complexes may exist as distinct proteins or as a single multidomain protein. The PTS catalyzes the uptake of carbohydrates and their conversion into their respective phosphoesters during transport. There are four successive phosphoryl transfers in the PTS. Initial autophosphorylation of EI, using PEP as a substrate, is followed by transfer of the phosphoryl group from EI to HPr. EIIA catalyzes the self-phosphoryl transfer from HPr after which the phosphoryl group is transferred to histidine or cysteine residues of EIIB. The sugar is transported through the membrane-bound EIIC and is phosphorylated by the appropriate sugar-specific EIIB.

|  |  |  |
| --- | --- | --- |
| Reference pathway | 184% 150% 122% 100% 82% 67% 55% | 图片下载 |
